# IL11-mediated stromal cell activation may not be the master regulator of pro-fibrotic signaling downstream of TGFβ

**DOI:** 10.3389/fimmu.2024.1293883

**Published:** 2024-02-22

**Authors:** Yunhao Tan, Kenta Mosallanejad, Qingxiu Zhang, Stephen O’Brien, Meghan Clements, Stuart Perper, Sarah Wilson, Sudiksha Chaulagain, Jing Wang, Mary Abdalla, Helen Al-Saidi, Danyal Butt, Anca Clabbers, Kwasi Ofori, Beth Dillon, Bohdan Harvey, John Memmott, Christopher Negron, David Winarta, Catherine Tan, Amlan Biswas, Feng Dong, Vanessa Morales-Tirado, Xiaoqing Lu, Gurminder Singh, Michael White, Shanna Ashley, Heather Knight, Susan Westmoreland, Lucy Phillips, Tracy Carr, Lauren Reinke-Breen, Rajeeva Singh, Jianwen Xu, Kan Wu, Lisa Rinaldi, Brian Stoll, Yupeng David He, Lisa Hazelwood, Jozsef Karman, Andrew McCluskey, William Stine, Ivan Correia, Stephen Gauld, Marc C. Levesque, Geertruida Veldman, Cedric Hubeau, Timothy Radstake, Ramkrishna Sadhukhan, Edda Fiebiger

**Affiliations:** ^1^ AbbVie Cambridge Research Center, Cambridge, MA, United States; ^2^ AbbVie Bioresearch Center, Worcester, MA, United States; ^3^ AbbVie Inc., North Chicago, IL, United States

**Keywords:** IL11, fibrosis, drug target, STAT3, ERK, stromal cell, signaling

## Abstract

Fibrotic diseases, such as idiopathic pulmonary fibrosis (IPF) and systemic scleroderma (SSc), are commonly associated with high morbidity and mortality, thereby representing a significant unmet medical need. Interleukin 11 (IL11)-mediated cell activation has been identified as a central mechanism for promoting fibrosis downstream of TGFβ. IL11 signaling has recently been reported to promote fibroblast-to-myofibroblast transition, thus leading to various pro-fibrotic phenotypic changes. We confirmed increased mRNA expression of IL11 and IL11Rα in fibrotic diseases by OMICs approaches and *in situ* hybridization. However, the vital role of IL11 as a driver for fibrosis was not recapitulated. While induction of IL11 secretion was observed downstream of TGFβ signaling in human lung fibroblasts and epithelial cells, the cellular responses induced by IL11 was quantitatively and qualitatively inferior to that of TGFβ at the transcriptional and translational levels. IL11 blocking antibodies inhibited IL11Rα-proximal STAT3 activation but failed to block TGFβ-induced profibrotic signals. In summary, our results challenge the concept of IL11 blockade as a strategy for providing transformative treatment for fibrosis.

## Introduction

Fibrosis is defined as the pathological accumulation of extracellular matrix (ECM) proteins and can occur in the context of chronic inflammation or impaired wound healing (1, 2). Dysfunction of multiple pathways that guide normal healing and wound repair can lead to irreversible tissue scaring rather than reconstitution of normal tissue architecture (1, 2). In autoimmune settings, fibrosis frequently results in a progressive pathology, which culminates in anatomical alterations, loss of tissue function(s), and organ failure (1, 2). The detrimental clinical manifestations of fibrosis highlight a pressing need in the development of innovative and transformative therapies to mitigate or even reverse fibrosis.

TGFβ has been identified as a central driver of the pathology of fibrosis (3). TGFβ cannot be targeted directly for therapeutic purposes in humans because of its broad biological functions and high toxicity risk (4). A substantial body of literature spanning human cell-based assays and murine *in vivo* models, describes IL11 as the master effector molecule of fibrosis development downstream of TGFβ and other pro-fibrotic factors (5–15). IL11 is a member of the IL6 cytokine family and is minimally expressed in healthy tissues (16). Comparative expression analysis demonstrated that IL11 transcripts are significantly upregulated in patients with fibrotic diseases such as cardiac fibrosis or Idiopathic Pulmonary Fibrosis (IPF) (5, 6). In IPF patients, IL11 expression levels and disease progression correlate inversely implying the close relevance of this cytokine and lung fibrosis (6). IL11 mRNA levels are also increased in the affected tissue of patients with inflammatory bowel disease (IBD) and upregulation of this cytokine is found most pronounced in patients with intestinal fibrosis (17, 18). Because of its function as a stimulator of bone marrow stem cell proliferation and maturation, recombinant IL11 was used to treat thrombocytopenia during chemotherapy (19, 20). This therapy was withdrawn from market because induction of fibrosis was observed as a side effect (20, 21).

Murine models of fibrosis with IL11- or IL11Rα-deficient and IL11-inducible animals revealed the pro-fibrotic nature of the IL11 signaling (7). Upon induction of cytokine production, IL11-inducible strains rapidly developed fibrosis in multiple organs (7). In contrast, IL11 deficient strains were protected from fibrosis development in several murine fibrosis models, including the bleomycin models of lung fibrosis and cardiac fibrosis (5, 6). IL11 expression pattern is low or undetectable in healthy murine tissues, but high in inflammation settings, such as acute DSS insult in the gut (18, 22). Support for an anti-IL11 therapy in the fibrosis space is based on the collective observations that IL11 blockade could reduce tissue inflammation and even reverse established fibrosis in various animal models of fibrosis (6, 12, 15, 18, 23, 24).

IL11 engages its cognate receptors and mediates downstream cellular responses via three distinct mechanisms (25). The “classic signaling” is initiated when the cytokine binds to the transmembrane IL11Rα chain, which permits interaction of the ligand-binding receptor subunit to the transmembrane co-receptor gp130 dimer. This trimer then dimerizes and forms the functional hexameric signaling complex which activates multiple transcription factors (25). “Trans signaling” occurs when IL11 binds to soluble IL11Rα, which consecutively engages the membrane-expressed gp130. In this scenario, soluble IL11Rα is generated via proteolytic cleavage by the metalloprotease such as ADAM10 (26). Cell line-based studies with the designer cytokine “hyper-IL11” (an IL11-IL11Rα fusion protein) demonstrated the potential of IL11 trans signaling to occur in recipient cells that solely express gp130. However, the existence of this pathway *in vivo* and its precise biological relevance in homeostasis and disease still warrant further research (25). The third signaling option, “trans-presentation”, occurs when IL11 binds to the transmembrane IL11Rα on a “transmitting cell” and engages gp130 on the surface of an adjacent cell, which leads to IL11 signaling activation (25). Similar to “trans signaling”, the physiological relevance of IL11 “trans-presentation” remains to be elucidated (25). Irrespectively of the type of activation, the primary IL11Rα proximal signaling pathways include the signal transducer and activator of transcription 3 (STAT3) and the extracellular signal-regulated kinase (ERK) (25). Although distinct mechanisms can be used by IL11 to engage IL11Rα and activate downstream signaling, our study focused on the “classic IL11 signaling” to monitor cellular responses to IL11 and effects of IL11 signaling blockade. Furthermore, it is plausible that antibodies interrupting the IL11-IL11Rα interaction at the critical interface (site I) (27), identified by structural-functional analysis, can block all forms of IL11 signaling regardless of their precise biological functions.

Our study sought to further elucidate the pro-fibrotic signaling aspects of IL11 biology, specifically its production, intracellular signaling, and effects on primary human fibroblasts. When comparing IL11 to TGFβ side by side, IL11 appears to be an unlikely candidate for being the main driver of pro-fibrotic signaling at the cellular level.

## Materials and methods

### Animal procedures

All experiments that involved live animals including naïve C57Bl/6 mice and the bleomycin (BLM) model of fibrosis were approved by the Institutional Animal Care and Use Committee of AbbVie. The bleomycin model of lung fibrosis was performed as described in the literature ( (6) with minor modifications similarly. Briefly, 2U/kg bleomycin (Meithal Pharma) or PBS (Invitrogen) was delivered directly to the lung via oropharyngeal aspiration to 8-week-old C57Bl/6 mice (Jackson Labs). Approximately 10 animals per bleomycin group were sacrificed at 7-, 14-, and 21-days post bleomycin delivery. Control PBS group was also sacrificed at 21 days. At takedown middle and post caval lobes were separated, stored in RNAlater (Invitrogen) and used for qRT-PCR. Superior and inferior lobes were used for hydroxyproline assay and performed according to manufactures instructions (Quickzyme). Left lobe was inflated with 10% neutral buffered formalin, tied off, removed, and immersed in the same fixative. The lobe was embedded in paraffin, sectioned at 5 microns and stained with H&E and Masson’s Trichrome.

### Human tissue

Formalin-fixed paraffin embedded (FFPE) tissue for histological evaluation was obtained from the University of Massachusetts Medical School, Worcester, Massachusetts. The study was approved by the Institutional Review Board of University of Massachusetts School of Medicine. Written informed consent was obtained from all donors. For this study, tissue was analyzed from IPF, Scleroderma, and IBD patients. IBD patients underwent surgical resection and, for controls, biopsies of the unaffected adjacent tissue to the resection were used. Tissues were also acquired from commercial vendors (Folio, Discovery Life Sciences, and Celerity Biosciences).

### Histology analysis, immunohistochemistry, and *in situ* hybridization

All FFPE blocks were sectioned at 5 µm in Diethyl pyrocarbonate (DEPC)-treated water. *In situ* hybridization was performed on the Leica Bond RX automated immunostainer using Advanced Cell Diagnostic’s (ACD/Bio-Techne, Newark, CA) RNAscope 2.5 LSx assay. ACD’s 2.5 LSx assay incorporates a series of designated reagents; epitope retrieval was done with both Leica’s Epitope Retrieval 2 (Cat #AR9640) and ACD’s Protease. Both human IL11 (Cat # 425288) and human IL11Rα (Cat # 568318) LSx probes were run, with a positive control probe, PPIB (Cat # 313908), and a negative control probe, dapB (Cat # 312038). All blocks were screened with PPIB prior to running with either human IL11 or IL11Rα to ensure proper mRNA integrity. Visualization was done with either ACD’s RNAscope brown LS reagent kit (Cat #322100) or the red LS reagent kit (Cat #322150). The slides were scanned on the Pannoramic 250 whole slide digital scanner (3DHISTECH Ltd, Budapest, Hungary) using a 40x lens with extended focus. Expression patterns were analyzed by a pathologist and samples were semi-quantitatively scored in multiple high-power fields for frequency of IL11 or IL11Rα positive cells with the following criteria: 0 = no IL11+ cells, 1 = rare cells, 2 = approximately 1-5% of cells, 3 = approximately 10-20% of cells, 4 = >20% of cells.

### Computational analysis of publicly available RNA-seq data set

We downloaded and re-processed the RNA-seq (GSE134692) (28) data from Gene Expression Omnibus, which reported pathways uniquely associated with end-stage IPF patients lung samples. We used ArrayStudio (Qiagen) to reprocess the downloaded data and to generate normalized gene expression matrices. Finally, the R package ggplot2 was used to generate figures depicting IL11 and IL11Rα gene expression.

### Production of recombinant proteins and antibodies

Human IL11 and mouse IL11 were expressed in E. coli and purified from soluble fractions by nickel affinity column followed by size exclusion chromatography (>98% purity by SDS-PAGE and HPLC analysis). The anti-IL11 mAb (5A6.2, mu IgG1/k) which can bind both human and mouse IL11, was produced in house and purified from the CHO cell clone (3E7).

### Binding assay using surface plasmon resonance technology

Binding kinetics of IL-11 antibody for recombinant human and mouse IL-11 was determined by SPR at 25°C using an anti-Fc capture assay. Briefly, IL-11 antibody was captured on an anti-human or anti-mouse Fc chip. Recombinant IL-11 (human or mouse), ranging in concentrations from 36 nM to 0.15 nM via a set of 3-fold dilution series, was injected over the reference and test the surface for 5 minutes at 50µl/minute. The data was fitted globally to a 1:1 binding model using Biacore T200 Evaluation software to determine the binding kinetic rate constants and *k_a_* (1/Ms) and *k_d_* (1/s) and equilibrium dissociation constant K_D_ (M).

### Cell culture

Primary normal human lung fibroblasts (NHLFs) were cultured in FGMTM-2 Fibroblast Growth Medium-2 Bullet Kit (Lonza). Human lung fibroblast lines (MRC-5 and IMR-90) and the human lung epithelial cell line (A549) were cultured in DMEM containing 10% FBS, penicillin and streptomycin (Pen+Strep), plus supplements of L-glutamine, sodium pyruvate, and non-essential amino acid (NEAA). RAW264.7 cells were cultured in complete RPMI. Cells were washed in PBS and detached from culture flasks with 5 ml Accutase.

To generate cell lines with stable transgene expression, lentiviral particles were purchased commercially (Sigma) or generated by the AbbVie lentiviral core facility. To enhance transduction efficiency, polybrene was added to the filtered supernatants (5 µg/ml) prior to transduction of A549 and RAW264.7 cells via spin-fection at 1250 x g for 60 min at room temperature. For establishing the pSTAT3 luciferase reporter RAW264.7 cell line, cells expressing the reporter construct were selected by puromycin (10 µg/ml), single cell cloning was performed to isolated stable clones via the standard limiting dilution approach.

### Gene expression, signaling pathways, and protein analyses

RNA was isolated from cell cultures using Qiashedder (Qiagen) and RNeasy Mini kits (Qiagan). Purified RNA was analyzed for gene expression on a CFX384 real time cycler (Bio-rad) using TaqMan Kit (Applied Biosystems) with indicated probes.

For Nanostring analysis, NHLFs from two independent donors (Donor 1: Lonza C2512, Lot-18TL052524; Donor 2: Lonza C2512, Lot-18TL057581) were stimulated with TGFβ and IL11 for indicated period, RNA was isolated, profibrotic gene expression analysis was performed by Canopy Biosciences using the nCounter fibrosis panel. NSolver version 4.0 (Nanostring) was used to process and normalize Nanostring data. We calculated combined ratio of treatments as indicated to the control samples (geometric mean of ratios for each donor).

For western blot analysis NHLF, A549, IMR90, and RAW264.7 cells (0.5x10^6^ per well) were seeded in 12 well plates and starved in low serum media (0.1% FBS) for 16-18 h. Next, cells were stimulated with cytokines for indicated periods, and subsequently lysed in 300 μl 1xLDS buffer containing TCEP (25 mM). Lysates were incubated at 65°C for 15 min. Before SDS-PAGE separation, lysates were passed through a BD 1 ml sub-Q syringe attached to a 26G needle to reduce viscosity. 15 μl of individual samples (15-20 µg protein from whole cell extract) were separated by SDS-PAGE followed by western analysis.

For secreted protein measurements, NHLFs were plated in 96 well plates (0.5 x10^5^ per well) and serum-starved in low serum media (0.1% FBS) for 16-18 h. Next, cells were stimulated with cytokines for indicated time periods. Supernatants were collected and stored at -80°C for future analysis, The remaining cells were lysed with the proprietary lysis buffer from the αSMA HTRF kits following the manufacturer’s instruction. Intracellular αSMA levels were measured with the EnVision plate reader (EX. 320 nm/EM. 620 nm).

ELISA assays were performed to measure secreted CTGF, TIMP1, IL6, and IL11. Cell culture supernatants or murine lung tissue homogenates (with protease inhibitor) were cleared of cell debris by spinning 96 well plates at 400 x g for 5 min. Supernatants were transferred to new 96 well plates. Concentrations of indicated analytes were measured following the manufacturer’s protocols.

### High content imaging of primary human lung fibroblasts

For αSMA and Collagen 1A1 quantification after cytokine stimulation, NHLFs were plated in 96 well BD Biocoat black walled clear bottom plates. (5 x10^3^ per well) and serum-starved in low serum media (0.1% FBS) for 16-18 h. Cells were then stimulated with cytokines for 24 h and fixed with formaldehyde (2% final concentration) at room temperature for 10 min. 0.1%. Triton X100 in PBS was used for permeabilization of fixed cells. 6% normal goat serum in PBS was used as the blocking reagent. For staining, primary antibodies and secondary antibodies were diluted with 3% normal goat serum in PBS. The antibody incubation period for both primary and secondary antibody staining was 60 min at room temperature. PBS was used for all washing steps. Images (20X) were taken with the Thermo Fisher Scientific Cell Insight CX7 High Content Analysis Platform. Quantification of the αSMA stress fibers was done using the cytoskeletal rearrangement assay tool within the HCS Studio software. The cytoskeletal rearrangement assay tool is enabled to detect actin fibers that are typically an organized series of “spots”. The identification of these organized spots is determined by length to width ratios (how long and wide the spots are) and perimeter to area (irregular or regular shape). The output of these parameters is determined by counting spots, evaluating the area and intensity of spots within the region of interest (ROI). Quantification of the Collagen 1A1 images was done using the compartmental analysis within the HCS Studio software. Spots are identified and intensity measured within two separate regions of interest using Circ (nucleus) and Ring (cell body) masks. Image quantification was performed using quantified % high total intensity within the ring region of interest (ROI).

### pSTAT3 luciferase reporter assay and endogenous STAT3 activation assay

To quantify the biological activity of IL11, RAW267.4 were seeded in 96 well plates (1x 10^5^ per well) and serum-starved overnight. The next day, serial dilutions of IL11 in serum free media were used to stimulate the reporter cells. To quantify the efficacy of the anti-IL11 mAb (5A6.2), serial 3-fold dilutions of anti-IL11 mAbs (ranging in concentrations from 680 nM to 0.03 nM) were pre-incubated with IL11 (1 nM) in serum free medium containing 0.1% BSA. To allow antibody-cytokine complex formation, samples were incubated at 37°C for 1 h before adding to the reporter cells. 5 h post stimulation, supernatants were discarded, and cells were lysed with the assay buffer from Nano-Glo^®^ Luciferase Assay System. Luciferase activity (implied for STAT3 activation levels) in the lysates were quantified by the MicroBeta2^®^ Microplate Counter.

To quantify endogenous IL11 signaling in NHLFs, cells were seeded in 96 well plates (1x 10^5^ per well) and serum-starved overnight. The next day, serial dilutions of IL11 in serum free media were used to stimulate NHLFs. To quantify the blocking efficacy of anti-IL11 mAb (5A6.2), indicated doses of 5A6.2 were preincubated with IL11 (20 ng/ml) for 1 h before added to NHLFs. IL11-mediatd STAT3 activation was quantified by the pSTAT3 HTRF kit from PerkinElmer following the manufacturer’s instructions.

### Effect of anti-IL11 antibody on TGFβ-mediated NHLF activation

To monitor the effect of the anti-IL11 antibody 5A6 on fibroblast activation induced by TGFβ, cells were seeded in 96 well plates (1x 10^5^ per well) and serum starved overnight. NHLFs were stimulated with TGFβ (5 ng/ml) for 24 hours in the presence of control IgG (low: 25 μg/ml, high: 50 μg/ml) or 5A6.2 (low: 25 μg/ml, high: 50 μg/ml). Supernatants were collected and assayed for the protein levels of TIMP1, CTGF, and IL6.

### Statistical analysis

Statistical significance for experiments with more than two groups tested with One Way Anova and Tukey multiple comparison tests. When comparisons between only two variables were made, unpaired two tailed t-test was used to assess statistical significance. Adjusted p-values were calculated with Prism7 (Graphpad) or with Excel. Asterisk coding, also indicated in figure legends, is depicted as follows: *: P <= 0.05; **: P <= 0.01; ***: P <= 0,001; ****: P <= 0,0001. Data presented are representative of at least 3 independent repeats unless otherwise designated. Data with error bars are represented as mean ± SEM.

## Results

### OMICs analysis and *in situ* hybridization studies confirmed upregulation of IL11 mRNA transcripts in multiple fibrotic diseases

**Figure 1:**
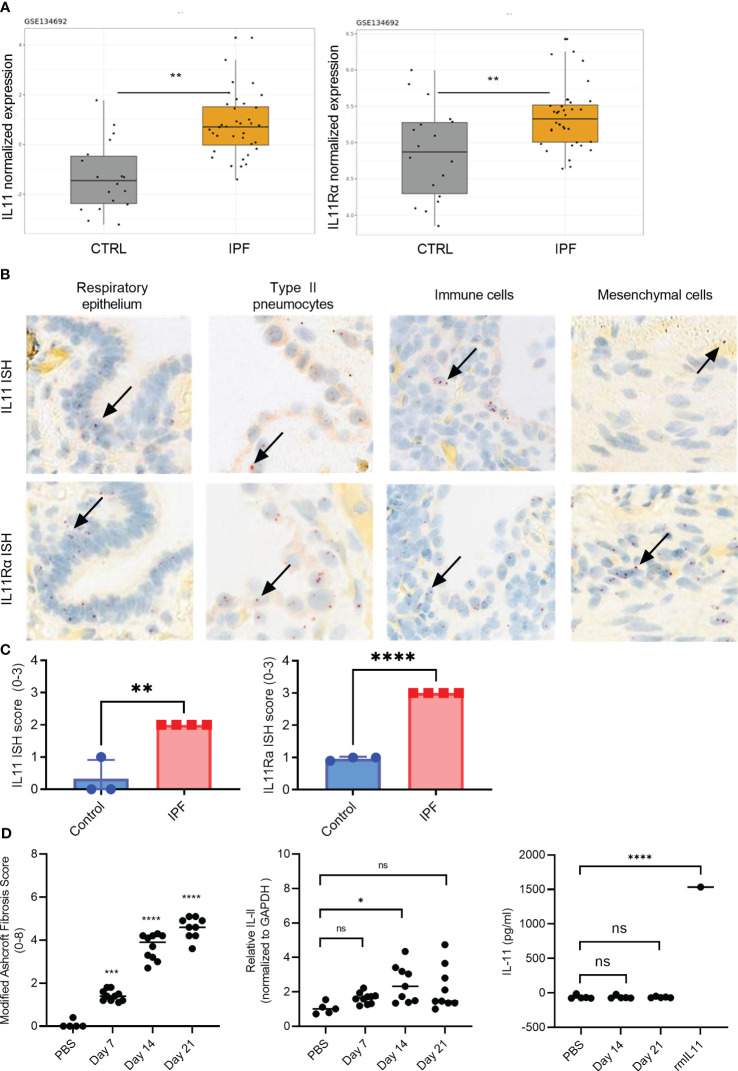
IL11 and IL11Rα mRNA levels are upregulated in the IPF and scleroderma. **(A)**. Normalized expression levels of IL11 mRNA (left) and IL11Rα mRNA (right) comparing healthy control and IPF patients. Computational analysis of the publicly available data set GSE134692. **(B)**. Representative IL11 and IL11 Rα RNA *in situ* (RNA ISH) hybridization images of IPF lung tissues. IL11 and IL11 Rα ISH signal (red dots) was detected in epithelial cells, type II pneumocytes, mesenchymal cells. The highest level of mRNA is detected in immune cells that are morphologically consistent with myeloid cells. Selected IL11 mRNA positive cells were marked by black arrows. *P ≤ 0.05; **P ≤ 0.01; ***P ≤ 0.001. **(C)**. Semi-quantitative scoring of IL11 (left) and IL11Rα (right) ISH in IPF (n=4) and normal lung tissues (n=3), with the following coring guide: 0: no positive cells; 1: Rare cells; 2: Roughly 1-5% of cells; 3: Roughly 10-20% of cells; 4: >25% of cells. **(D)**. Time course analysis of the murine model of bleomycin-induced lung fibrosis (day 7-, 14-, and 21-post bleomycin injury). Modified Ashcroft fibrosis score (Grades 0-8) was used to quantify the degree of fibrosis in mouse lung tissues (left). qPCR analysis of IL11 mRNA expression levels from mouse lung homogenates collected post bleomycin treatment on day 7, day 14, and day 21 post bleomycin injury (middle). IL11 protein expression analysis from the lung tissue homogenates was performed by ELISA on day 7, day 14, and day 21 post bleomycin. 1.5 ng (1500 pg) of recombinant mouse IL11 (mIL11) was used as positive control (right). Representative data of at least 2 independent experiments. P-values were determined by one-way ANOVA or Student’s t-tests. *P ≤ 0.05; **P ≤ 0.01; ***P ≤ 0.001; **** P ≤ 0.0001; ns, not significant.

We combined computational and histological approaches to validate the expression status of IL11 and IL11Rα in human fibrotic and additional autoimmune diseases. For computational analysis, we downloaded and re-processed the RNA-seq data (GSE134692) (28). Internal analysis via the ArrayStudio platform was performed to reprocess the dataset and to generate normalized gene expression matrices. Subsequently, IL11 gene expression was visualized using the R package ggplot2. These serial data analysis workflows revealed that IL11 and its receptor expression were upregulated in IPF lungs (n = 36) when compared to controls (n = 19) in bulk RNA-seq data (p<0.01) (**Figure 1A**). In summary, the RNA expression pattern shows that IL11 expression is elevated in IPF patients with end-stage disease progression.

To gain a more detailed understanding of the cell types that expressed IL11 in IPF, we next performed a set of *in situ* hybridization studies (ISH) (**Figure 1**). Analysis of IL11 and IL11Rα in consecutive sections of lung tissue samples confirmed increased expression in IPF compared to controls. Most consistently, the IL11 mRNA signal was detected in epithelial cells and type II pneumocytes. Based on nuclear morphology, a myeloid cell type in IPF lungs displayed high IL11 mRNA expression. Mesenchymal cells consistently expressed higher levels of IL11Rα than IL11. Overall, IL11Rα was expressed by multiple cell types including airway epithelial cells, pneumocytes and type II pneumocytes, fibroblasts and myofibroblasts, pleural mesothelial cells, adipocytes, and immune cells (**Figure 1B**). Semi-quantitative scoring of IL11 and IL11Rα ISH in the control (n = 3) and IPF lung tissues (n = 4) revealed significantly higher IL11 and IL11Rα expression in disease tissues in comparison to control lungs (**Figure 1C**, **Figure S1**).

**Figure 2:**
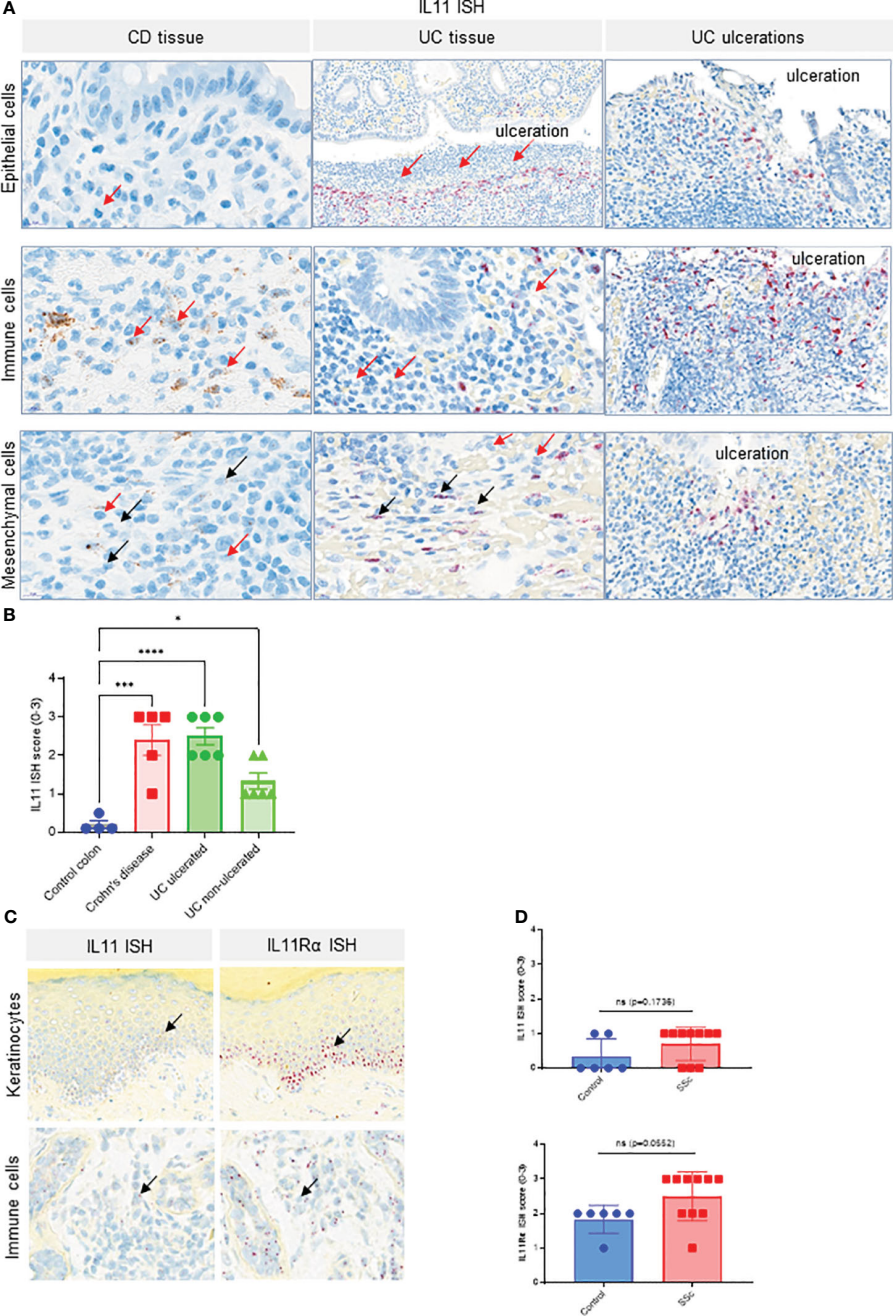
IL11 mRNA is upregulated in colon tissue of CD and UC patients. **(A)**. Representative IL11 RNA *in situ* hybridization (ISH) images of the colonic mucosa of CD and UC samples. Cell types were classified based on their nuclear morphology. Detection of IL11 mRNA in immune cells (including neutrophils) in the lamina propria particularly in the proximity to ulcerations (red arrows), intestinal epithelial cells, and mesenchymal cells (fibroblasts, smooth muscle cells). IL11 mRNA positive cells were marked by black arrows. Neutrophils were marked by red arrows. **(B)**. Semi-quantitative scoring of IL11 ISH in mucosal samples (control (n=4), CD (n=5), and ulcerated UC (n=6) and non-ulcerated UC (n=6)), with the following coring guide: 0: no positive cells; 1: Rare cells; 2: Roughly 1-5% of cells; 3: Roughly 10-20% of cells; 4: >25% of cells. **(C)**. Representative IL11 and IL11Rα RNA ISH images of systemic sclerosis (SSc) skin samples. IL11 and IL11Rα mRNA expression was detected in keratinocytes of the basal layer, spinosum, and in immune cells. IL11 mRNA positive cells were marked by black arrows, **(D)**. Semi-quantitative scoring of IL11 ISH (upper) and IL11Rα ISH (lower) in systemic sclerosis (SSc) skin samples [control (n=6), SSc (n=10)], with the following scoring guide: 0: no positive cells; 1: Rare cells; 2: Roughly 1-5% of cells; 3: Roughly 10-20% of cells; 4: >25% of cells. P-values were determined by one-way ANOVA or Student’s t-tests. *P ≤ 0.05; ***P ≤ 0.001; **** P ≤ 0.0001; ns, not significant.

We also confirmed published reports of elevated IL11 expression in inflammatory bowel disease (IBD). IL11 ISH studies in Crohn’s disease (CD) and Ulcerative Colitis (UC) patient colon resection samples showed significantly higher transcript levels compared to control colon tissue (**Figure 2A**, **Figure S1**). Lower IL11 expression was seen in non-ulcerated UC than in ulcerated UC samples. IL11-positive cells were most prevalent adjacent to ulcerations and in proximity to infiltrating neutrophils. The highest level of IL11 expression was found in infiltrating large immune cells, morphologically consistent with myeloid cells. Low expression was found in epithelial cells and mesenchymal cells, with morphologies consistent with fibroblasts and smooth muscle cells, located adjacent to ulcerations (**Figure 2A**). Semi-quantitative scoring of IL11 ISH in the mucosa of controls (n = 4), CD (n = 5), ulcerated UC (n = 6), and non-ulcerated UC (n = 6) yielded significantly higher IL11 expression in CD and ulcerated UC and little to no expression in control colons (**Figure 2B**, **Figure S1**). In serial sections from normal skin samples, little to no IL11 ISH signal was detected, but sporadic IL11Rα ISH signals were found, particularly in the epidermis of normal skin (**Figure S1B**). Using the same approach, we observed a trend that IL11 and its receptor transcripts were expressed in systemic sclerosis (SSc) patient skin keratinocytes with sporadic signals detected in infiltrated immune cells in SSc patient skin samples (**Figure 2C**). When quantifying IL11 and IL11Rα expression using ISH scores, we failed to detect statistically significant increase of IL11 or IL11Rα transcripts in SSc patient skin samples (n = 10) in comparison to normal skin samples (n = 6) (**Figure 2D**). Collectively, these observations suggested that IL11 mRNA elevation is only associated with IPF and IBD (UC and CD).

We next sought to determine whether the observed IL11 mRNA elevation in human disease settings can be corroborated by the murine model of bleomycin-induced pulmonary fibrosis. Although the Ashcroft score from the lung tissues of the bleomycin-treated mice increased (**Figure 1D**), which is indicative of lung fibrosis development, no statistically significant increase in IL11 mRNA levels was observed during the early (day 7) or late stages (day 21) of bleomycin administration (**Figure 1D**). A modest increase of IL11 mRNA levels was detected at the day 14 (**Figure 1D**). Irrespectively, no IL11 protein was detected by ELISA in mouse lung tissue homogenates from day 14 and day 21 samples (**Figure 1D**). Since we failed to detect any IL11 protein expression in the murine bleomycin model, we deemed further animal experiments to block IL11 protein inappropriate and focused on interrogating the IL11 signaling pathways and functional consequences of IL11 signaling blockade using *in vitro* human cell culture models.

### Pro-inflammatory and pro-fibrotic mediators drive IL11 secretion

**Figure 3:**
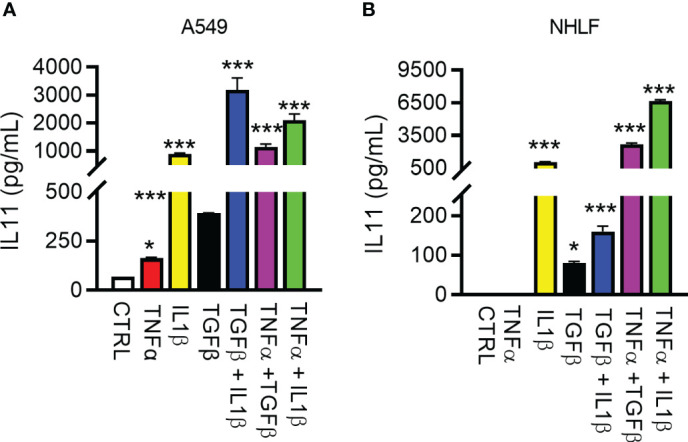
Regulation of IL11 protein by pro-inflammatory and pro-fibrotic cytokines in human lung epithelial cells and fibroblasts. **(A)**. Secreted IL11 in the supernatant of the human lung epithelial cell line (A549 cells) was quantified by ELISA after treatment with TGFβ, IL1β, TNFα, and cytokine combinations. The following cytokine concentrations were used for 24h-treatment: TNFα, 5 ng/ml; IL1β, 5 ng/ml; TGFβ, 5 ng/ml; TGFβ + IL1β, 2.5 ng/ml each; TGF + TNFα, 2.5 ng/ml each; IL1β + TNFα, 2.5 ng/ml each. **(B)**. IL11 in the supernatant of the cultured primary human lung fibroblasts was quantified by ELISA (TNFα, 5 ng/ml; IL1β, 5 ng/ml; TGFβ, 5 ng/ml; TGFβ+IL1β, 2.5 ng/ml each; TGFβ +TNFα, 2.5 ng/ml each; IL1β + TNFα, 2.5 ng/ml each; all cytokine treatment lasted for 24 h). Representative data of at least 2 independent experiments. P-values were determined by one-way ANOVA or Student’s t-tests. *P ≤ 0.05; ***P ≤ 0.001, Bar graphs represent means and standard deviations of two independent experiments.

Detailed studies on IL11 induction *in vitro* have been performed with the goal of improving the understanding of upstream regulators of IL11 and the cellular sources of this cytokine in fibrotic diseases. Early studies reported several cytokines relevant to fibrosis and inflammation induces IL11 in epithelial cells (29). We thus used the human lung epithelial cell line A549 and primary normal human lung fibroblasts (NHLFs) to elucidate factor(s) that regulates IL11 production. The inflammatory stimulus TNFα - induced low levels of IL11 in A549s, but not in NHLFs, in contrast to IL1β and TGF1β (**Figures 3A, B**). In side-by-side comparisons, IL1β was significantly more potent in inducing IL11 than TGFβ. Furthermore, an additive effect of IL11 induction was noted when A549 cells or NHLF were treated with cytokine combinations (**Figures 3A, B**). Together, these data suggested that the profibrotic cytokine (TGFβ) and proinflammatory cytokines (TNFα and IL1β) individually and additively promote IL11 production from human lung epithelial cells and fibroblasts.

### IL11-mediated cell activation induces pSTAT3 and pERK activity in cell lines and primary fibroblasts

**Figure 4:**
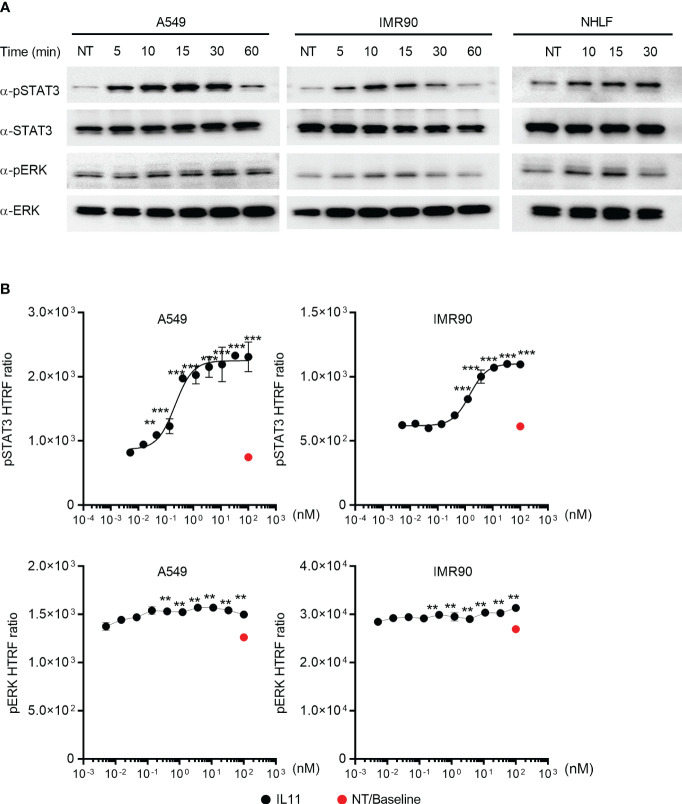
IL11 stimulation induces STAT3 and ERK phosphorylation in human lung epithelial cells and fibroblasts. **(A)**. Indicated human cell lines and primary cells were stimulated with IL11 (10 ng/ml) for indicated time periods (A549, left; IMR90, middle; primary human lung fibroblasts, right) and lysed. Phosphorylated and total protein levels of STAT3 and ERK were detected by Western blotting analysis. Representative blots from at least two independent experiments are shown. **(B)**. Quantification of IL11-mediated STAT3 and ERK phosphorylation in A549 and IMR90 cell lines via homogeneous time-resolved fluorescence (HTRF). Cells were stimulated with indicated doses of IL11 (top concentration: 100 nM, with 3-fold serial dilution, bottom concentration: 0.005 nM), STAT3 and ERK phosphorylation were quantified by HTRF. Representative of at least 2 independent experiments. P-values were determined by one-way ANOVA or Student’s t-tests. **P ≤ 0.01; ***P ≤ 0.001.

To determine the intracellular signaling cascades induced by IL11, we focused on monitoring STAT3 and ERK, as they are commonly activated by the IL6 superfamily cytokines (16). IL11 stimulation of the human lung epithelial cell line A549 and the human lung fibroblast cell line IMR90 induced STAT3 and ERK phosphorylation (**Figure 4A**). The same signaling activity was also observed with primary human lung fibroblasts (NHLFs) (**Figure 4A**). We next sought to quantify IL11-mediated STAT3 and ERK activation via HTRF (Homogeneous Time Resolved Fluorescence). IL11 promoted STAT3 phosphorylation in a dose-dependent manner (**Figure 4B**). In contrast, a binary activation pattern was observed for ERK phosphorylation, in which similar levels of ERK phosphorylation were detected with most IL11 concentrations (**Figure 4B**). Whereas some recent reports claimed that IL11 preferentially engages the ERK pathway (5, 6), our data demonstrated that IL11 clearly induces both STAT3 and ERK signaling in human lung epithelial cells and fibroblasts and that STAT3 phosphorylation serves as a more sensitive marker for IL11 signaling initiation.

### IL11 is dispensable for fibroblast activation *in vitro*

**Figure 5:**
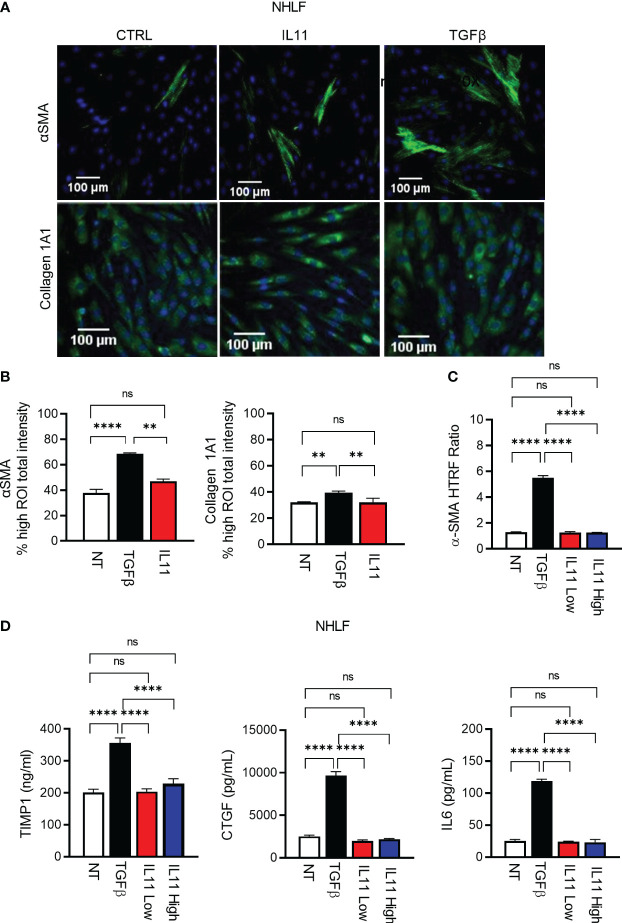
IL11 alone is not sufficient to trigger fibroblast activation *in vitro* in comparison to TGFβ. **(A)**. Selected fluorescence images of αSMA and Collagen 1A1 immunostaining in primary human lung fibroblasts treated with indicated cytokines (TGFβ, 10 ng/ml; IL11, 10 ng/ml; All cytokine treatment lasted for 24 h; scale bar 100 µm; magnification 20 x); αSMA ROI was quantified using cytoskeletal rearrangement assay tool from the HCS Studio software. Collagen 1A1 ROI was quantified using the compartmental analysis from the HCS Studio software. Details regarding ROI quantification are included in the methods section. **(B)**. Quantification of fluorescence intensity of αSMA and Collagen 1A1 immunostaining in primary human lung fibroblasts treated with indicated cytokines (TGFβ, 10 ng/ml; IL11, 10 ng/ml; All cytokine treatment lasted for 24 h). Representative of 2 independent experiments, mean and SD of 3 technical replicates are depicted. **(C)**. Quantification of intracellular αSMA protein expression by HTRF in primary human lung fibroblasts treated with indicated cytokines (TGFβ, 10 ng/ml; IL11 low, 10 ng/ml; IL11 high, 100 ng/ml; All cytokine treatment lasted for 72 h). **(D)**. Quantification of secreted pro-fibrotic and pro-inflammatory mediators (TIMP1, left; CTGF, middle; IL6, right) from primary human lung fibroblasts treated with indicated cytokines by ELISA (TGFβ, 10 ng/ml; IL11 low, 10 ng/ml; IL11 high, 100 ng/ml; All cytokine treatment lasted for 72 h). Representative of at least 2 independent experiments. P-values were determined by one-way ANOVA or Student’s t-tests. **P ≤ 0.01; **** P ≤ 0.0001; ns, not significant. Bar graphs represent means and standard deviations of two independent experiments.

Using high content imaging analysis to quantitatively measure human fibroblast activation, we compared IL11- and TGFβ-mediated fibroblast activation with the fibrosis biomarkers αSMA (**Figure 5A**, **Figure S2A**) and Collagen 1A1 as readouts (**Figure 5A**, **Figure S2B**). In investigator-selected visual fields, both cytokines appeared to induce αSMA and Collagen 1A1 production in primary human lung fibroblasts (**Figure 5A**, **Figure S2**). However, an unbiased quantitative analysis of regions of interest (ROI) from image composites (**Figure S2**) demonstrated that IL11 failed to induce these protein markers in a statistically significant manner (**Figure 5B**). In contrast, TGFβ induced Collagen 1A1 and αSMA protein production remained statistically significant in the ROI analysis (**Figure 5B**). In parallel with the high content imaging approach, we monitored cytokine-induced αSMA protein accumulation via HTRF. Whereas TGFβ induced robust αSMA protein production (**Figure 5C**), we were still unable to detect any induction of αSMA in IL11 treated cells by HTRF, regardless of varying the doses of IL11 (IL11 low: 10 ng/ml; IL11 high: 100 ng/ml) and extending the cytokine treatment period to 72 hours. Likewise, TGFβ induced release of the ECM modifiers (TIMP1 and CTGF) and the pro-inflammatory cytokine (IL6) from NHLFs (**Figure 5D**). Notably, IL11 stimulation failed to induce these markers at the protein level in a statistically significant manner (**Figure 5D**). Collectively, these results highlighted that TGFβ potently promotes fibroblast activation *in vitro*, whereas IL11 alone fails to activate fibroblasts comparably.

### Antibody-mediated IL11 neutralization blocks STAT3 activation but fails to mitigate pro-fibrotic cellular processes induced by TGFβ 

**Figure 6:**
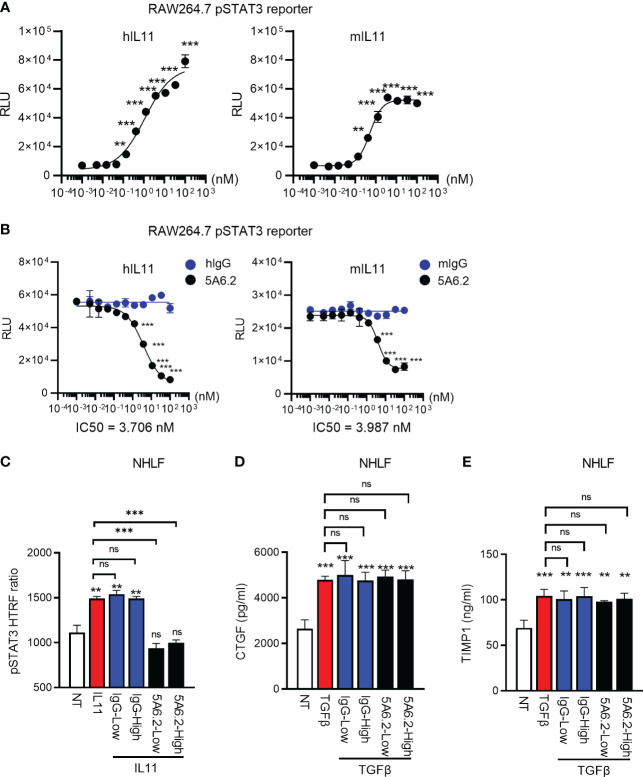
Anti-IL11 antibody blocks IL11Ra-proximal STAT3 activation but fails to affect TGFβ-mediated fibroblast activation. **(A)**. Quantification of IL11-mediated STAT3 activation via the RAW264.7 pSTAT3 luciferase reporter assay. Cells were stimulated with indicated doses of IL11 (top concentration: 100 nM, with 3-fold serial dilution, bottom concentration: 0.005 nM, all cytokine treatment lasted for 24 h), STAT3 phosphorylation levels were quantified by the Nano-Glo luciferase assay system. **(B)**. Quantification of IL11 neutralization efficacy by the anti-IL11 antibody 5A6.2 via the RAW264.7 pSTAT3 luciferase reporter assay. Cells were stimulated with IL11 alone (1 nM) or the IL11-antibody complex formed by preincubating IL11 (1 nM) with indicated concentrations of the anti-IL11 antibody 5A6.2 (top concentration: 682 nM, with 3-fold serial dilution, bottom concentration: 0.03 nM; All cytokine treatment lasted for 24 h), STAT3 phosphorylation levels were quantified by the Nano-Glo luciferase assay system. **(C)**. Quantification of IL11 neutralization efficacy by the anti-IL11 antibody 5A6.2 in primary human lung fibroblast via the pSTAT3 HTRF assay. Cells were stimulated with IL11 alone (1 nM) or the IL11-antibody complex formed by preincubating IL11 (1 nM) with indicated concentrations of the anti-IL11 antibody or IgG control (5A6.2 high and IgG high, 50 μg/ml; 5A6.2 low and IgG low, 25 μg/ml; All treatment lasted for 24 h). **(D, E)**. ELISA of secreted TIMP1 and CTGF from primary human lung fibroblasts treated with TGFβ1 (5 ng/ml, 24h) in the presence of the anti-IL11 antibody 5A6.2 or IgG control (5A6.2 high and IgG high, 50 μg/ml; 5A6.2 low and IgG low, 25 μg/ml). Cell culture supernatants were collected 24 h post stimulation for ELISA analysis. Representative of at least 2 independent experiments. P-values were determined by one-way ANOVA or Student’s t-tests. **P ≤ 0.01; ***P ≤ 0.001.

We next developed an anti-IL11 tool antibody 5A6.2 in house to block IL11 signaling. Based on Biacore kinetics analysis, 5A6.2 is a high affinity anti-IL11 antibody with single digit nanomolar dissociation constant (K_D_) against recombinant human IL11 and mouse IL11 (**Table S1**). The biophysical binding characteristics of 5A6 are comparable to that of the anti-IL11 mAb X203 reported in the literature (6). Because of our observation that STAT3 is activated by IL11 in human lung epithelial cells and fibroblasts, we developed a luciferase-based pSTAT3 reporter assay to characterize the internally generated antibody 5A6.2 (**Figures 6A, B**). IL11 stimulation induced an increase in pSTAT3 luciferase reporter activity in a dose dependent manner and the luciferase signal was dose dependently reduced by 5A6.2 (**Figures 6A, B**).

IL11 has been proposed as a central effector molecule downstream of TGFβ, which subsequently propels the pro-fibrotic program in stromal cells (5–7). Therefore, we sought to determine the effect of 5A6.2 on TGFβ-mediated primary human lung fibroblast (NHLF) activation. In comparison to IgG controls, 5A6.2 expectedly inhibited STAT3 activation induced by IL11 (**Figure 6C**). However, in the context of TGFβ-mediated fibroblast activation, 5A6.2 failed to inhibit TIMP1 and CTGF release induced by TGFβ in NHLFs regardless of the antibody doses tested (Low: 25 µg/ml; High: 50 µg/ml; **Figures 6D, E**). This observation implies that inhibition of IL11 signaling is not sufficient to mitigate primary human lung fibroblast activation induced by the master pro-fibrotic cytokine TGFβ.

### IL11 failed to induce a pro-fibrotic gene signature comparable to TGFβ in primary human lung fibroblasts

**Figure 7:**
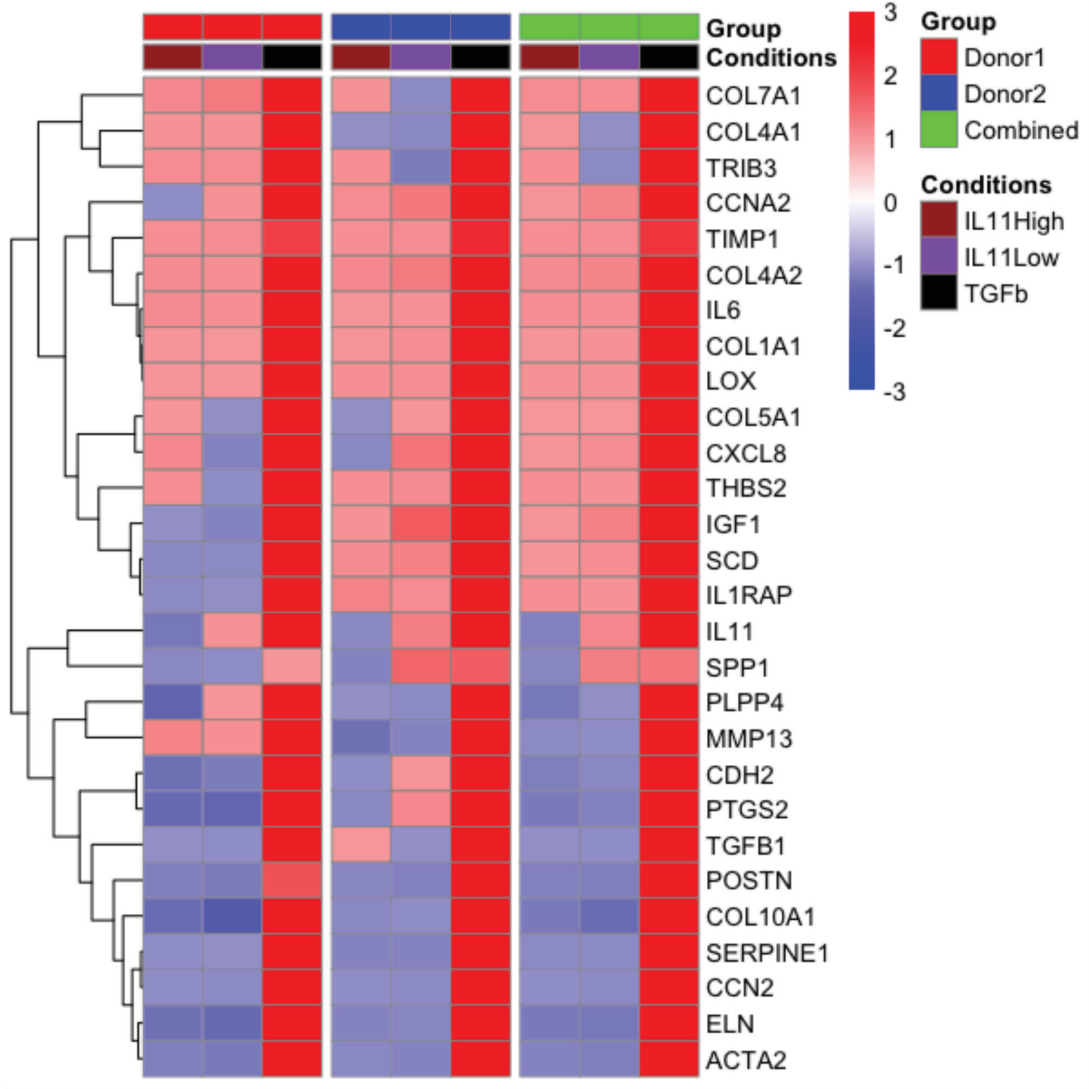
The pro-fibrotic gene signature induced by IL11 is quantitatively and qualitatively inferior to that induced by TGFβ in primary human lung fibroblasts. . Representative comparison of pro-fibrotic gene signature in IL11 and TGFβ stimulated primary human lung fibroblasts. Cells were stimulated with indicated cytokines (TGFβ, 5 ng/ml; IL11 high, 100 ng/ml; IL11 low, 10 ng/ml) for 24 h. RNA was extracted and subjected to Nanostring analysis using the nCounter fibrosis panel. Plots of geometric mean of expression ratios (normalized to control samples) of significantly upregulated genes (top 25) relative to TGFβ stimulation was shown. Primary human lung fibroblasts from 2 donors were used for the nanostring nCounter analysis. NSolver version 4.0 (Nanostring) was used to process and normalize Nanostring data. Combined ratio of treatments was calculated based on the levels of transcripts detected from the control samples (geometric mean of ratios for each donor), thus the results shown have been normalized to control.

To further investigate the functional consequences of IL11 signaling in fibrosis without bias, we performed nCounter analysis in primary human lung fibroblasts stimulated with TGFβ (5 ng/ml) and two doses of IL11 (IL11 Low: 10 ng/ml; IL11 High: 100 ng/ml) for 24 h. From the nCounter fibrosis panel, we observed that TGFβ stimulation induced robust mRNA expression of pro-fibrotic markers, including IL11 (**Figure 7**). Notably, a subset of profibrotic transcripts were upregulated upon IL11 stimulation in a statistically significant manner (geometric mean normalized to unstimulated control cells). IL11 treatment modestly induced the transcription of mediators of fibrotic processes including ECM components (Col1A1, Col4A1, Col4A2, Col7A1), ECM modifiers (TIMP1, THBS2 and LOX), stress responses proteins (TRIB3), growth factor (IGF1), inflammatory mediators (IL6, CXCL8, and IL1RAP), metabolism (SCD), and cell cycle regulation (CCNA1) (**Figure 7**). The IL11-dependent gene signature in our study is represented by CCNA2, Col1A1, Col4A2, TIMP1, and LOX, which were deconvoluted from 2 donors. The top 5 genes induced by TGFβ were COL7A1, COL4A1, TRIB3, CCNA2, and TIMP1. The expression profile confirmed our earlier observations that the TGFβ-induced profibrotic transcription landscape is superior to that of IL11 in both quantitative and qualitive manners. We further notice a disconnect between gene transcription and protein synthesis. For instance, while TIMP1 and IL6 transcript numbers were elevated by IL11 treatment, we consistently failed to detect the proteins in tissue culture supernatants (**Figure 5D**).

## Discussion

Recent literature supports the therapeutic hypothesis that antibody-mediated blockade of IL11 will prevent the onset of and even reverse tissue fibrosis (5, 6, 12, 13, 24, 30). Preventing IL11 from binding to and activating its receptor should break the vicious circle of disease progression in fibrosis. Our observations demonstrated that IL11 indeed activates a sub-set of TGFβ effector molecules at the mRNA level, but quantitatively the pro-fibrotic gene signature induced by IL11 signaling appears to be much inferior to that of TGFβ. Likewise, IL11 failed to induce the production of several classic pro-fibrotic markers (e.g. Collagen 1A1, αSMA, CTGF, TIMP1, and IL6) at the protein level when compared to TGFβ treatment side-by-side in multiple *in vitro* cell-based assays. Our results thus challenge the concept of IL11 as a master driver of fibrosis and imply that blocking IL11 will unlikely suffice to prevent or reverse fibrosis in the presence of TGFβ.

Our study did confirm that IL11 transcripts were low in healthy tissues while conversely increased in multiple fibrotic diseases. Significant upregulation of IL11 mRNA in tissues collected from patients with distinct fibrotic diseases was also recently reported (6, 11, 17, 18, 22). The cell types with a strong IL11 ISH signal were immune cell infiltrates, and lower expression was observed in epithelial and endothelial cells.

Based on recent publications, strong IL11 expression in fibroblasts was observed in CD and ulcerated UC patient tissue adjacent to ulcerations, while fibroblasts only occasionally expressed IL11 mRNA in IPF and SSc (6, 11, 17). Whereas IL11Rα showed a ubiquitous expression pattern in healthy tissue (25). In line with this, we did observe an increase of IL11 and mRNA transcripts IL11Rα in IPF patient lung tissues as well as IL11 mRNA elevation in UC and CD patient colon tissues. In contrast, the correlation between SSc and IL11/IL11Rα expression levels requires additional investigation. It is noteworthy that multiple attempts to demonstrate IL11 or its receptor at the protein levels in human disease tissues failed. This observation can be explained by the quality issues of commercially available IHC grade antibody, or by the common disconnection between mRNA abundancy and protein translation (31), or both. As such, future exploratory efforts are warranted to develop sensitive, reliable reagents, and approaches to monitor the protein levels of IL11 and its receptor in diseased/fibrotic human tissues.

Intriguingly, our time course analysis of mouse lung tissues collected after 7-, 14-, and 21-day post bleomycin treatment only revealed a modest elevation of IL11 transcripts at the 14-day time point, whereas we failed to capture any significant release of IL11 protein from the murine lung tissue homogenates. These observations could potentially be explained by the following scenarios. First, after bleomycin treatment, the temporal pattern of IL11 mRNA induction may misalign with our assay time points. Second, IL11 release in the mouse lung tissue could be transient and local, followed by rapid receptor engagement and internalization, thereby precluding any “excessive” IL11 protein release detectable by ELISA. We speculate that while below the detection threshold of the experimental approaches used, IL11 production at the local tissue environmental might contribute to the development of murine lung fibrosis induced by bleomycin. Irrespectively, the results from the *in vivo* murine bleomycin model prompted us to focus on further validation of IL11 biology using human cell culture models.

Multiple recent publications with human and murine cell-based assay platforms proposed IL11 as a central profibrotic driver *in vitro* (5, 6, 11, 12). Using several different experimental strategies, we were not able to observe statistically significant induction of classic pro-inflammatory and pro-fibrotic mediators at the protein level by IL11 stimulation *in vitro* (e.g. Collagen 1A1, αSMA, TIMP1, CTGF, and IL6). Regarding to IL11 signaling blockade strategies, the anti-IL11 antibody X203 has been used in the literature to block or even reverse fibrosis markers *in vitro* (in the context of TGFβ-mediated stromal cell activation) and fibrosis development in various pre-clinical *in vivo* fibrosis models (6, 12). We generated 5A6.2, a monoclonal anti-IL11 antibody, which is similar to the characteristics of the reported anti-IL11 antibody X203 (6). Despite validating the efficacy of 5A6.2 to block IL11 signaling at the receptor proximal level via the pSTAT3 luciferase assay and direct monitoring of STAT3 phosphorylation in primary human lung fibroblasts, this antibody failed to block TGFβ-mediated fibroblast activation *in vitro*. Our observation suggests that while TGFβ potently induces IL11 production, IL11 *per se* exerts limited impact on the overall pro-fibrotic programs induced by TGFβ signaling.

We acknowledge that there are several limitations associated with the current studies. Much of the profibrotic functions of IL11 was attributed to its involvement in the post-transcriptional signaling processes (5). In keeping with this, most of the fibrosis and inflammation mRNA markers induced by IL11 stimulation were modest compared to unstimulated controls and far inferior to that of TGFβ stimulation in primary human lung fibroblasts. However, when examining the effect of IL11 on the production of classic pro-fibrotic and pro-inflammatory markers (e.g. TIMP1, CTGF, IL6, and αSMA) at the protein level, we consistently failed to detect the increase of these markers from IL11-stimulated cells when compared to cells stimulated with TGFβ. It is possible that IL11 would induce a more profound change in the global proteomic scale, which is not captured by the classic profibrotic markers utilized in our study. Thus, exploration of the IL11-induced secretome is warranted in future studies to provide a “holistic insight” into the human stromal cell responses to this cytokine. In addition, whereas structure biology insights suggested that antibody-mediated blockade of IL11 site I as a feasible strategy to disrupt IL11-IL11Rα binding (25), we have not explored strategies that target other sites of the IL11-IL11Rα interfaces. Furthermore, targeting IL11Rα was reported to be a viable strategy to mitigate fibrosis development *in vitro* and *in vivo* (12, 24, 30). Such a strategy has been pursued by our industry peers (e.g. Lassen Therapeutics). Our current study did not provide a direct comparison between IL11-targeting and IL11Rα-targeting strategies. Lastly, while we detected IL11 expression in myeloid cells in the lung tissue, how IL11 regulates the reciprocal signaling crosstalk between immune cells and stromal cells and its implication in fibrosis would require further investigation in co-cultured models.

In summary, our observations extend the understanding of IL11 biology with a focus on stromal cells and highlight an auxiliary, rather than a driving role, of this cytokine in fibrosis. We generated an IL-11 blocking antibody (5A6.2) that was able to block IL11 signaling at the receptor proximal level but failed to ameliorate the fibrosis-relevant readouts *in vitro*. Our results imply that IL11 may be a useful biomarker for fibrotic and inflammatory human diseases due to its expression pattern. Meanwhile, our data establish a mandate to re-evaluate the effect of IL11 on fibroblast activation and the therapeutic concept of reversing fibrosis via antibody mediated IL11 neutralization.

## Data availability statement

The datasets presented in this study can be found in online repositories. The names of the repository/repositories and accession number(s) can be found in the article/**Supplementary Material**.

## Ethics statement

Ethical approval was not required for the studies on humans in accordance with the local legislation and institutional requirements because only commercially available established cell lines were used. The animal study was approved by Institutional Animal Care and Use Committee. The study was conducted in accordance with the local legislation and institutional requirements.

## Author contributions

YT: Conceptualization, Data curation, Investigation, Project administration, Writing – original draft, Writing – review & editing, Supervision. KM: Data curation, Writing – review & editing. QZ: Data curation, Writing – review & editing. SO’B: Data curation, Writing – review & editing. MC: Writing – review & editing. SP: Data curation, Writing – review & editing. SWi: Data curation, Writing – review & editing. SC: Data curation, Writing – review & editing. JW: Writing – review & editing. MA: Data curation, Writing – review & editing. HA-S: Data curation, Writing – review & editing. DB: Data curation, Writing – review & editing. AC: Data curation, Writing – review & editing. KO: Data curation, Writing – review & editing. BD: Data curation, Writing – review & editing. BH: Data curation, Writing – review & editing. JM: Data curation, Writing – review & editing. CN: Data curation, Writing – review & editing. DW: Data curation, Writing – review & editing. CT: Data curation, Writing – review & editing. AB: Data curation, Writing – review & editing. FD: Data curation, Writing – review & editing. VM-T: Writing – review & editing. XL: Data curation, Writing – review & editing. GS: Data curation, Writing – review & editing. MW: Data curation, Writing – review & editing. SA: Data curation, Writing – review & editing. HK: Data curation, Writing – review & editing. SWe: Data curation, Writing – review & editing. LP: Data curation, Writing – review & editing. TC: Data curation, Writing – review & editing. LR-B: Data curation, Writing – review & editing. RSi: Data curation, Writing – review & editing. JX: Data curation, Writing – review & editing. KW: Data curation, Writing – review & editing. LR: Data curation, Writing – review & editing. BS: Data curation, Writing – review & editing. YH: Data curation, Writing – review & editing. LH: Data curation, Writing – review & editing. JK: Data curation, Writing – review & editing. AM: Data curation, Writing – review & editing. WS: Data curation, Writing – review & editing. IC: Data curation, Writing – review & editing. SG: Data curation, Writing – review & editing. ML: Data curation, Writing – review & editing. GV: Data curation, Writing – review & editing. CH: Writing – review & editing. TR: Resources, Supervision, Writing – review & editing. RSa: Conceptualization, Investigation, Project administration, Resources, Supervision, Writing – review & editing. EF: Conceptualization, Investigation, Project administration, Resources, Supervision, Writing – original draft, Writing – review & editing.

## Supplementary material

Supplementary Figure 1 IL11 and IL11Rα mRNA expression levels are low in normal lung, colon tissue, and skin tissues (related to , ). **(A)**. Representative IL11 ISH images in serial sections cut from normal lung (n=3), colon (n=3), and skin tissues (n=3). **(B).** Representative IL11 Rα ISH images in serial sections cut from normal lung(n=3), colon(n=3), and skin tissues (n=3); IL11Rα mRNA positive cells were marked by black arrows.

Supplementary Figure 2 High content imaging composites for αSMA and Collagen 1A1 immunostaining in cytokine treated primary human lung fibroblasts (related to ). **(A)**. Collagen 1A1 immunostaining image composite for primary human lung fibroblasts treated with indicated cytokines (TGFβ, 10 ng/ml; IL11, 10 ng/ml; All cytokine treatment lasted for 24 h; 20x). Red boxes highlight selected fields presented in . Representative images of at least 3 independent experiments are shown. **(B)**. αSMA immunostaining image composite in primary human lung fibroblasts treated with indicated cytokines (TGFβ, 10 ng/ml; IL11, 10 ng/ml; All cytokine treatment lasted for 24 h; 20x). Red boxes highlight selected fields presented in ; Representative images of at least 3 independent experiments are shown.
