# Supplementary figures and images for: IL11-mediated stromal cell activation may not be the master regulator of pro-fibrotic signaling downstream of TGFβ
Source: Front Immunol. 2024 Feb 22;15:1293883. doi: 10.3389/fimmu.2024.1293883 (PMC10917968; doi:10.3389/fimmu.2024.1293883)

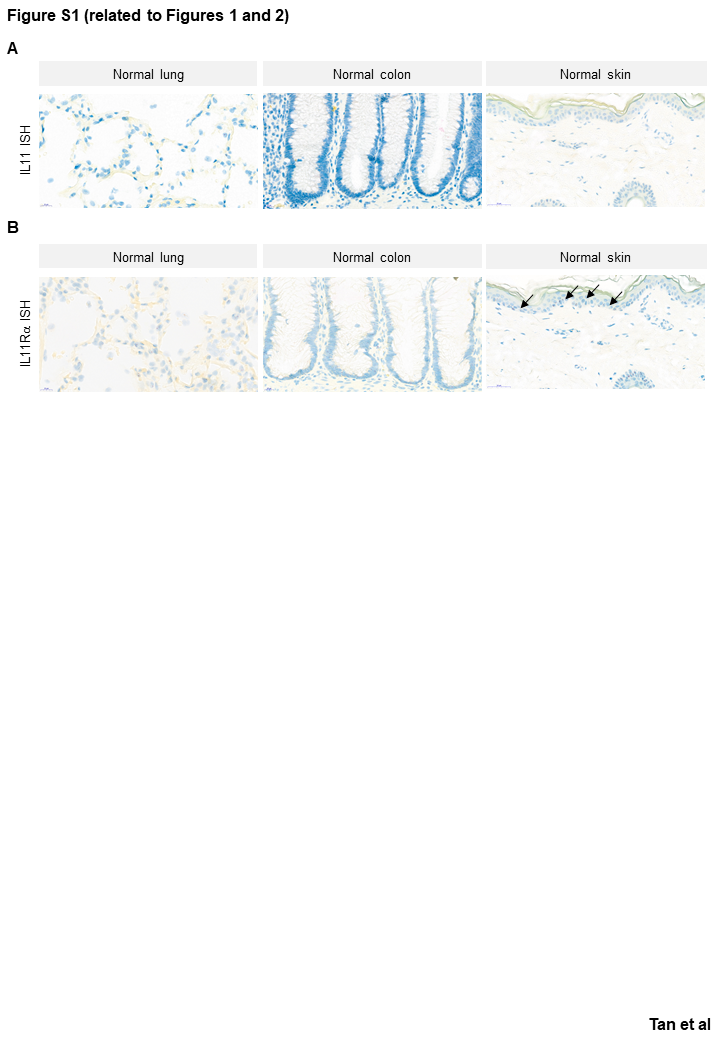

Supplement: Supplementary Figure 1 — IL11 and IL11Rα mRNA expression levels are low in normal lung, colon tissue, and skin tissues (related to , ). (A). Representative IL11 ISH images in serial sections cut from normal lung (n=3), colon (n=3), and skin tissues (n=3). (B). Representative IL11 Rα ISH images in serial sections cut from normal lung(n=3), colon(n=3), and skin tissues (n=3); IL11Rα mRNA positive cells were marked by black arrows. [file Image_1.tif]

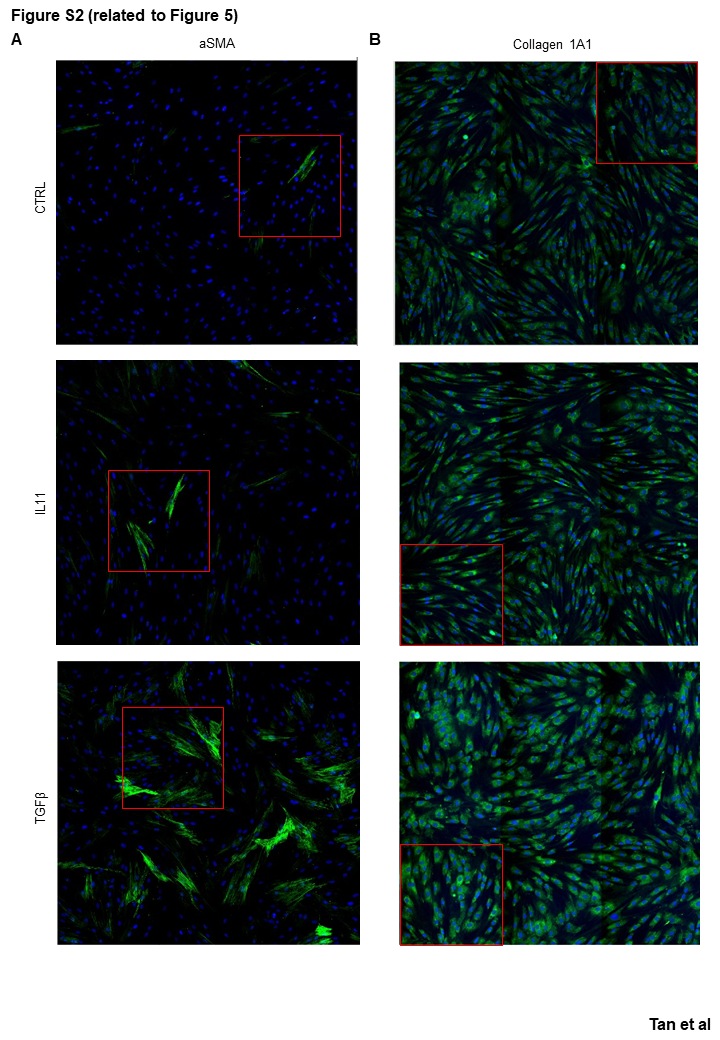

Supplement: Supplementary Figure 2 — High content imaging composites for αSMA and Collagen 1A1 immunostaining in cytokine treated primary human lung fibroblasts (related to ). (A). Collagen 1A1 immunostaining image composite for primary human lung fibroblasts treated with indicated cytokines (TGFβ, 10 ng/ml; IL11, 10 ng/ml; All cytokine treatment lasted for 24 h; 20x). Red boxes highlight selected fields presented in . Representative images of at least 3 independent experiments are shown. (B). αSMA immunostaining image composite in primary human lung fibroblasts treated with indicated cytokines (TGFβ, 10 ng/ml; IL11, 10 ng/ml; All cytokine treatment lasted for 24 h; 20x). Red boxes highlight selected fields presented in ; Representative images of at least 3 independent experiments are shown. [file Image_2.tif]
