# Supplementary material for: IL11-mediated stromal cell activation may not be the master regulator of pro-fibrotic signaling downstream of TGFβ
Source: Front Immunol. 2024 Feb 22;15:1293883. doi: 10.3389/fimmu.2024.1293883 (PMC10917968; doi:10.3389/fimmu.2024.1293883)
Supplement: Supplementary file 3 [file DataSheet_1.pdf]

Table S1

Human IL-11			Mouse IL-11		
k_a(1/Ms)	k_d(1/s)	K_D (M)	k_a(1/Ms)	k_d(1/s)	K_D (M)
$3.5x10^5$	$1.4x10^{-3}$	$3.9x10^{-9}$	$1.2x10^5$	$2.1x10^{-3}$	$1.7x10^{-9}$
